# On the “Flu”—A Theory

**Published:** 1919-03

**Authors:** S. N. Young

**Affiliations:** Cincinnati, Ohio


### ON THE "FLU"—A THEORY

BY S. N. YOUNG, D.D.S., CINCINNATI, OHIO

In discussing the "flu," one layman remarked to another: "I do not believe there will be cessation of the epidemic until there occur severe electric storms to burn up the germs and purify the air;" which expression leads to the following ponderable question: The electric storm —the generator of what?

For more than four years active warfare had been in progress; the factor of explosives being confined chiefly to a given area. During the latter part of such warfare, nitrogenous explosives were used with apparent reckless abandon. From that environment, and during the final period, the epidemic originated.

To proceed with the thought: The approximate atmospheric content necessary for normal animation is well known—chiefly oxygen and nitrogen, one part to four; free oxygen being vital to metabolism, nitrogen (the control) being necessary when free in greater proportion, retarding respiration, and oxidation in fact.

The explosive used nowadays, while more or less of secret composition, as nitrogenous content, and upon action, liberates a certain percentage of free nitrogen; in quantity, proportionate, varying from the minute of the rifle cartridge to that most impressive quantity of the heaviest bomb and shell; the total freed, immense, incalculable.

What of the consequence! A very minute quantity of free nitrogen is utilized, in season, through the influence of bacteria, by the vegetable kingdom. Excess, disseminating, overwhelmes utility of free oxygen, interfering with the process of metabolism of animal life—a process of controlled oxidation.

This being true, environmental evidence should be manifest. And what do we find? Those energetically engaged in compelling oxygen activity to the utmost possibility, comparatively unaffected; and the lower animals, in the same environment, dying by thousands. Note the progress of the epidemic: By current, influenced by friction of rotation, south and north, then westward, progressively, to encompass the globe.

Given free nitrogen in excess, or rather an overpowered free oxygen, what should the pathological manifestation represent? Logically, a disorder suggesting suboxidation. Pursuing the allusion, what do we find? Conceding the minimum of vital depression imputable to fear, camp-shock, inferior food quality, and like factors; those afflicted, the suboxidized, or toxemic if you prefer, to whom full oxygen proportion is essential to hold a vascillating metabolic balance. Note grossly the semblance of the average victim, observable to even inexperienced readers; one enencumbered with the pathological superfluous, or, the pathological sub.

The acquistion of a condition of suboxidation, or toxemia, being understood, and atmospheric insufficiency being assumed, the preventive, or the remedy should suggest itself. Eliminate the toxemic condition in such manner as the degree of intensity indicates, and stabilize with proper habits—dietetic and otherwise. Urge a proper ptyalinization of food (when food is indicated), especially well the class which requires that digestive enzyme; avoiding combinations that will immediately antagonize a digestive ferment; also those, one class of which will excite, too soon, secretion of a ferment counter to a preactive ferment. Restrict the use of articles of common dietary questionable as a food. Prescribe the more oxygen-containing food, *i. e.*, natural food with content undestroyed or least injured in preparation, of class as conditions indicate; and individual fresh air, with physical exercise sufficient to establish and maintain oxygen equilibrium.

Note, in this connection, the harmony of the theory with accepted ruling of the health authorities; the prohibiting of congregations; fresh air, compelled to a verge of public ridicule; fruit juices and fresh fruits and vegetables prescribed ''to alkalinize the blood.''

As to the duration and recurrence. In the electric storm we have a generator of ozone (and of nitrogen in a utilizable compound). In sunshine-chlorophyll we have another natural factor bearing upon atmospheric equilibrium. Who knows how long time the restoration will require? Providence has been significantly kind, in so far as the sunshine-chlorophyll-animation is concerned, but the electric storms, unlike our fears and bad habits, are not with us always.

